# Leveraging Model Master Files for Long-Acting Injectables

**DOI:** 10.1007/s11095-025-03824-4

**Published:** 2025-01-28

**Authors:** Yuqing Gong, Robert Hopefl, Tonglei Li, Andrew C. Hooker, Daniela Amaral Silva, Khondoker Alam, Murray Ducharme, Rebecca Moody, Pratik Saha, Andrew Babiskin

**Affiliations:** 1 Division of Quantitative Methods and Modeling, Office of Research and Standards, Office of Generic Drugs, Center for Drug Evaluation and Research, U.S. Food and Drug Administration, 10903 New Hampshire Avenue, Silver Spring, , MD 20993 USA; 2 Department of Industrial and Physical Pharmacy, Purdue University, West Lafayette, IN USA; 3 Department of Pharmacy, Uppsala University, Uppsala, Sweden; 4 Simulations Plus, Inc, Lancaster, CA USA; 5 Learn and Confirm Inc, St. Laurent, QC Canada; 6 Office of Pharmaceutical Quality, Center for Drug Evaluation and Research, U.S. Food and Drug Administration, Silver Spring, MD USA; 7 GlaxoSmithKline, Collegeville, PA USA

**Keywords:** long acting injectables, model integrated evidence, model master file

## Abstract

The U.S. Food and Drug Administration (FDA) and the Center for Research on Complex Generics (CRCG) hosted a public workshop on May 2–3, 2024, titled “Considerations and Potential Regulatory Applications for a Model Master File”. The workshop aimed to discuss the application of the Model Master File (MMF) concept in regulatory submissions that contain model integrated evidence (MIE), improving model sharing, model standardization, regulatory consistency, and regulatory efficiency. On Day 1, there was a session dedicated to MMF applications for long-acting injectables (LAIs). This perspective summarizes presentations, panel discussion, and small group discussion for the potential applications of MMFs in LAI product development, including case studies and potential situations in which MMFs can support regulatory submissions. The scientific presentations discussed the application of MMFs in mechanistic physiologically based pharmacokinetic (PBPK), multiphysics simulation, and population pharmacokinetics (popPK) models, as well as the potential utility of a model-integrated bioequivalence (MI-BE) framework. Additionally, challenges and considerations of implementing MMFs for LAIs were discussed in the panel and small groups. The anticipated benefits of MMFs are recognized among model developers, industries, and regulators.

**Graphical Abstract:**

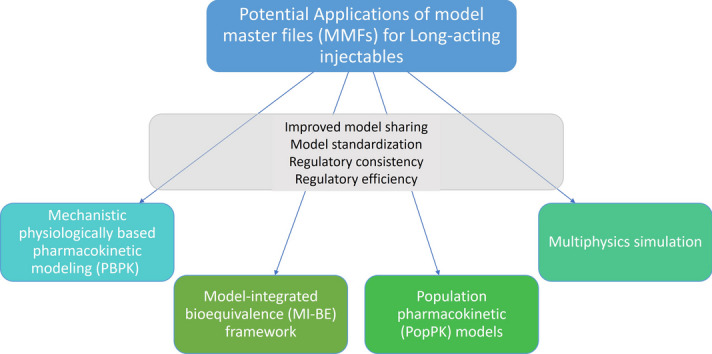

## Introduction

Long-acting injectables (LAIs) are considered an important alternative to oral medications for patients with chronic conditions such as schizophrenia, human immunodeficiency virus (HIV), and cancer, as they reduce dosing frequency and improve patient adherence [1–3]. The development of LAIs provides patients and healthcare providers with more treatment options. However, development of LAIs is a lengthy, complex, and costly process. Due to the long-acting nature of LAIs, a conventional bioequivalence (BE) study used to assess product equivalency or post-approval changes may take months or even years to complete [4]. Challenges of BE studies are mainly due to the long study duration, subject recruitment, potential high dropout rate, and increased variability that are associated with BE studies.

Model integrated evidence (MIE) has been increasingly used in the development and assessment of generic LAIs. Research has shown that MIE can be used to support alternative BE studies or generate pivotal evidence for BE decisions, such as investigating in vitro to in vivo extrapolation (IVIVE), and developing more efficient BE study designs with a shorter duration or reduced sample size through either population PK (popPK) or PBPK-based virtual bioequivalence (VBE) [4]. The Model Master File (MMF) concept has been introduced as a framework to increase model sharing and model reusability that can be used in regulatory submissions that contain MIE [5]. The concept as introduced envisioned an MMF as a regulatory submission that could be referenced across products and formulations by multiple applications, similar to the Drug Master File (DMF), thereby improving model reusability, regulatory consistency, and regulatory efficiency. To build a consensus on potential applications and best practices of utilizing MMFs, the U.S. Food and Drug Administration (FDA) organized a public workshop with the Center for Research on Complex Generics (CRCG) on May 2–3, 2024 [6]. This perspective summarizes presentations, panel discussion, and small group discussion for the potential applications of MMFs in LAI development in a session titled “MMF Applications for Long-Acting Injectable Drug Products”.

## Scientific Presentations

### Regulatory Utility of Mmf for Development Of Lais

*Andrew Babiskin, Ph.D., FDA*

Dr. Andrew Babiskin shared his insights into the challenges of BE studies for LAIs and how MIE and MMF can be useful to address some of those challenges. MIE is commonly used for two different strategies for alternative BE approaches in LAIs: (1) enhancing efficiency of pharmacokinetic (PK) BE studies through popPK modeling; and (2) BE based on in vitro characteristics/studies in lieu of PK BE studies supported by mechanistic PBPK modeling. Utilizing MIE for regulatory applications comes with the challenge of meeting regulatory standards; thus, sufficient verification and validation for the intended regulatory use is crucial [4]. Dr. Babiskin elaborated on the potential use cases of MIE approaches leveraging popPK models to reduce sample size and shorten duration of in vivo steady-state PK studies, which can be accomplished by simulating BE studies with virtual patients or simulating steady-state conditions from non-steady state conditions, respectively. These approaches have the potential to serve as MMFs to establish the appropriate methodology and evaluation that can be applied to multiple drug products. Additionally, Dr. Babiskin discussed the opportunity for mechanistic in vitro/in vivo correlation (IVIVC) models as MMFs for LAIs. The mechanistic IVIVC model can be developed by incorporating relevant local physiology, physicochemical properties of the active pharmaceutical ingredient (API), dissolution data, and particle size into the PBPK model, then validated using in vivo PK data of formulations with different release rates. The developed and validated IVIVC model could be reused for different drug products after revalidating the model by the applicant with their own formulations. Overall, the implementation of MMF provides several benefits, including supporting standardization in model verification and validation, facilitating MIE applications across different products, saving time in redeveloping similar methods, increasing applicant’s confidence in alternative study designs, and reducing regulatory assessment efforts across applications.

### MMF Application in Mechanistic IVIVCs for LAIs

*Daniela Silva, Ph.D., Simulations Plus, Inc*

Dr. Daniela Silva discussed the potential utility of MMF in the context of mechanistic IVIVCs. The development and regulatory evaluation of LAI products face challenges due to limited understanding about how and what product attributes and API characteristics affect tissue response and in vivo performance. PBPK modeling offers a distinctive approach to characterize interactions between LAI drug products and physiological processes. A case study on a low solubility compound was used to demonstrate the utility of PBPK modeling in the LAI space [7]. In this study, subcutaneous drug suspensions were injected in rabbits. Five different formulations that differed in particle size and in vitro dissolution were tested. The baseline PBPK model was calibrated against the intravenous plasma concentration vs. time (Cp-time) profile. Then, the PBPK model was assessed for whether it could link the LAI formulations to their in vivo exposure. The dissolution of the suspensions was mechanistically described taking into consideration physicochemical characteristics (e.g., particle size and solubility) and physiological parameters (e.g., depot volume). The study found that the effective in vivo particle size could be greater than the in vitro-measured data due to the aggregation of particles at injection site [8, 9]. Hence, the in vitro particle size distribution (PSD) was scaled to an in vivo PSD using the same scaling factor for all formulations. The Cp-time profiles were reasonably well described across all formulations. The scaling of PSD suggests that aggregation happens slowly over time. In addition, the deposition of foreign materials in the subcutaneous space can result in injection site reaction [10] and the inflammatory response can lead to a transitory increase in the depot volume [11–16], which can lead to a higher initial dissolution rate. As the inflammation subsides, the volume decreases and a more sustained drug release is obtained. The inclusion of inflammation in the model resulted in an improved prediction of the Cp-time profile shapes. The model was able to distinguish differences in exposure for all formulations and a combination of mechanisms was required to correctly describe the shape of the observed Cp-time profiles.

This case example demonstrated the potential application of MMF on mechanistic IVIVC models. For this type of application, the MMF would include information on the API (i.e., physicochemical and PK properties), physiological aspects in the platform (e.g., blood flow and tissue composition) as well as justification of the model structure (e.g., mechanisms impacting drug dissolution and absorption into systemic disposition). The MMF could also qualitatively inform the model development for similar formulations; however, parameters such as inflammation should be considered with caution, since different compounds and formulations can result in different magnitudes of inflammation.

### Multiphysics Simulation Framework for LAIs

*Tonglei Li, Ph.D., Purdue University*

**Fig. 1:**
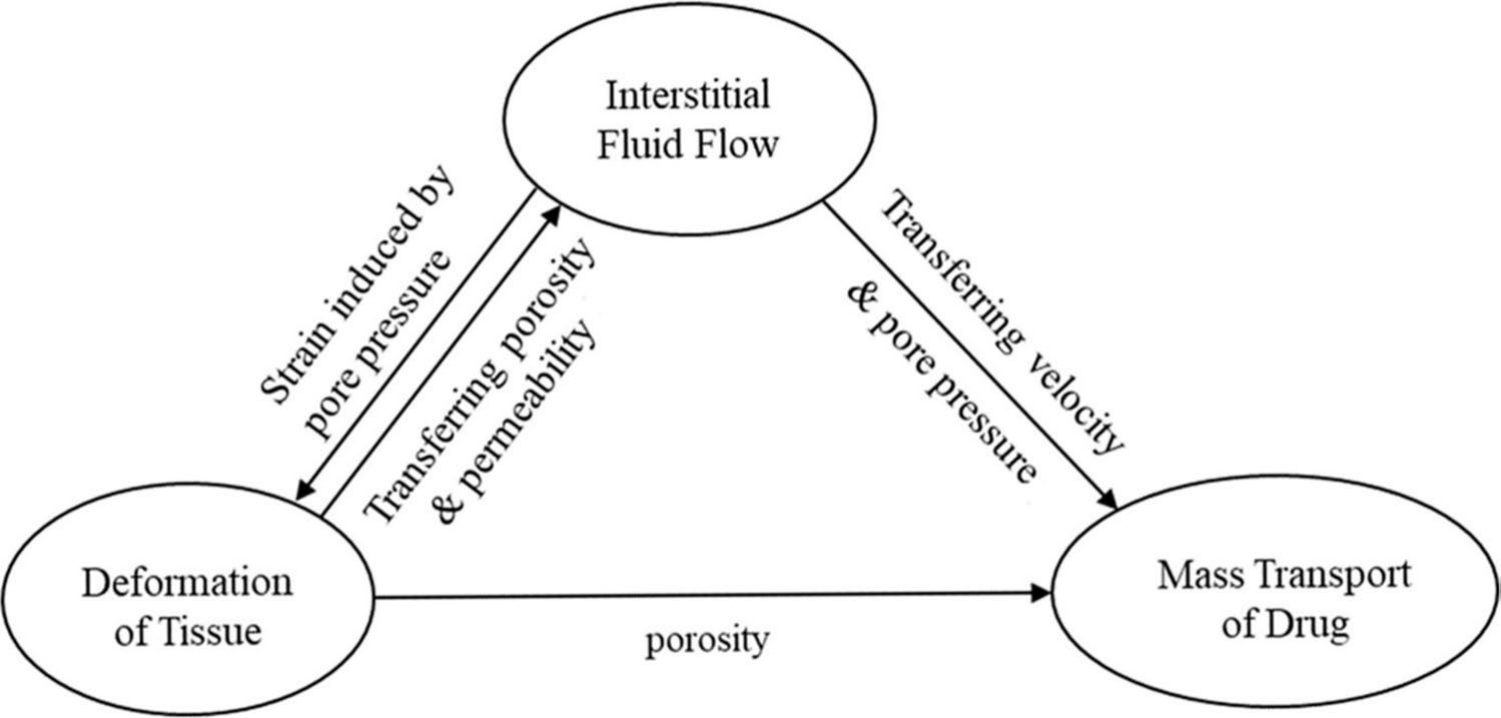
Coupling of tissue and drug transport properties in simulating drug transport and absorption at the local injection site (Adapted from Fig. 1c in Zheng, F et al. [17])

Dr. Tonglei Li presented the use of multiscale, multiphysics simulation to study the in vivo performance of drug delivery systems [17–23], including LAIs. Their studies were aimed to bridge critical quality attributes (CQAs) and systemic exposure of LAIs and locally injected delivery systems. The developed simulation framework provided practical implications in demonstrating the feasibility of using first principles to predict an injected product's transport/absorption kinetics at the injection site. Tissue properties, including those of interstitial fluid, could be fully coupled with diffusivity, membrane permeability, and partitioning attributes of the drug molecule, as well as release kinetics from the delivery vehicle (Fig. 1). The local computational fluid dynamics (CFD) could be solved numerically by well-established finite element methods. The multiphysics simulation was then integrated into a whole-body, compartment-based PK model to predict the product's systemic exposure. The systemic clearance and other PK parameters could be obtained based on clinical data of the IV injected product of the same drug molecule. Current results showed that multiphysics simulation permits sensitivity analyses of CQAs of LAI products on local and systemic exposure, which can be used to inform formulation design and product development and potentially guide regulatory review.

### Model-Integrated Bioequivalence (MI-BE) Methods Serving as MMF for LAIs

*Andrew Hooker, Ph.D. Uppsala University, Sweden*

Prof. Andrew Hooker presented MI-BE methods that could be used as MMF for LAIs. This MI-BE assessment method used popPK (i.e., nonlinear mixed-effect) models to assess BE and was developed under the Generic Drug User Fee Amendment (GDUFA) funded research program (HHSF223201710015C, 75F40119C10018).

Based on previous results, the MI-BE methods can control Type I error even for studies with small sample size and/or sparse sampling. They also exhibit higher power than the standard noncompartmental analysis (NCA)-based BE methods, especially as the data becomes sparser and/or more variable [24]. Further, model averaging MI-BE methods can be used to mitigate any model uncertainty that may be present [25].

The MI-BE methods are particularly powerful because they do not require standard study designs and can be applied to alternative, more efficient designs. For example, in the case of LAIs, BE testing in subjects typically necessitates a multiple-dose crossover steady-state design if using standard NCA-based BE analysis methods. Due to the long half-life of these drugs, this can result in lengthy BE study durations. However, an alternative switch study design can be employed with MI-BE analysis. In this design, BE is assessed by comparing the steady-state PK observations (i.e., drug concentrations) of the reference product with those of the test product during the first 1–2 dosing intervals after switching from the reference product [4]. PopPK models are used to fit the data, separate the superposition of the test and reference products post-switch, and then perform a MI-BE analysis. This approach avoids the imprecise task of determining when steady state occurs after switching treatments and can dramatically shorten study duration, albeit with the tradeoff of requiring more participants.

Implementation of MI-BE methods could be simplified through the use of an MMF. For example, having a way to share and qualify methods (i.e., the MMF) would aid in the practical implementation of the approach, and give more confidence to future users of the method. Further, with the implementation of specific popPK models in an MMF, that MMF could be used for other MI-BE analyses for a specific drug, patient population, and study design with less time needed for regulatory assessment. The benefits of model sharing in an MMF, especially public model sharing include: (a) improved reproducibility and validation of scientific results, (b) knowledge propagation: faster development of new science, (c) explicit evaluation and verification of models, and (d) model standardization.

## Panel Discussions

MMFs have potential to increase accessibility to modeling and simulation approaches to facilitate the development and lifecycle management of drug products. There is currently a lack of generic LAIs, and MIE may assist generic manufacturers to bring LAIs to the market more efficiently. The concepts of MIE and MMF can also support innovator pharmaceutical companies with post-approval changes of LAI products. The panel discussion focused on the potential applications and challenges of MMFs for LAI formulations. The panel was moderated by Drs. Yuqing Gong (FDA) and Pratik Saha (GlaxoSmithKline) and included speakers previously introduced in this article as well as Drs. Khondoker Alam (FDA), Murray Ducharme (Learn and Confirm Inc), and Rebecca Moody (FDA).

The panel first discussed the status of MIE in generic approvals for LAIs and the concerns and challenges of applying MMF for LAIs. Several generic LAIs have been approved, including the first three generic LAI microsphere products that were recently approved in 2023. Most applications utilized traditional approaches to assess BE. Dr. Andrew Babiskin commented that modeling and simulation has been used internally at the FDA for development of product specific guidances (PSGs) for LAIs. Innovative approaches like modeling and simulation can be helpful with accelerating LAI development and bringing more LAIs to market. Dr. Murray Ducharme commented that pursuing innovative approaches over standard approaches is often perceived as an additional risk for generic applicants. Thus, applicants often favor standard approaches to meet regulatory requirements. Dr. Babiskin noted that issues that may arise while using innovative approaches, may also occur when using standard approaches. Ultimately, a willing generic applicant needs to successfully utilize modeling and simulation for LAI development, so that other applicants will gain confidence in innovative approaches.

Compared to other dosage forms such as oral drugs, validation of LAI models is more complicated and challenging. There is an overwhelming benefit for applicants of having an MMF for LAIs. Dr. Murray Ducharme commented that he has experience with generic applicants attempting to use MIE for generic LAI development and it requires a significant amount of time and resources. If this work could be placed into an MMF, it would significantly decrease the cost of bringing generic LAIs to the market. Dr. Andrew Hooker noted that many of the newly approved innovator LAIs have used or incorporated popPK models in their drug development process and that there is a sufficient number of models developed in these new drug applications that could be utilized to investigate MIE approaches for generic LAIs.

Since there are different formulations of LAIs such as Poly Lactic-co-Glycolic Acid (PLGA), gels, and nano-suspensions, the panel discussed the scenario when an MMF may be used across different products and formulations. When administering the same API, the post-dissolution or disposition portion of the model could be translatable between drug products. If the mechanisms of release are different between products, then the MMF would need to be parameterized to that specific formulation. Dr. Daniela Silva gave the example that an MMF for a suspension formulation could not be directly applied to a PLGA-based product. The portion of the model describing mechanisms of release would need to be developed and validated to be applicable. Dr. Rebecca Moody commented that we need better understanding of what causes formulation variance for LAIs and better in vitro tools to properly describe these characteristics with PBPK models. Dr. Pratik Saha agreed that understanding the impact of formulation changes on PK performance is a significant challenge with LAIs; hence, development of in vitro tools to characterize the drug release from depot and imaging techniques to characterize the depot volume will be essential for developing the necessary inputs for mechanistic models of LAIs.

There was also discussion about whether MMFs would be open-source or confidential and how much information would be shared in an MMF. Some commentors expressed the view that MMFs submitted to the FDA should be kept confidential, but that MMF holders should not be prohibited from publishing or sharing their MMFs publicly. Panelists and audience members acknowledged that there are positive and negative aspects of both approaches, but the final decision will need to be determined by the MMF holder.

## Small Group Discussions

In person, small groups discussed potential benefits/incentives for stakeholders to develop and use an MMF for LAIs. The small discussion groups noted that the MMF framework would be particularly beneficial for LAI products, due to the length of in vitro and in vivo testing of these products and the overall cost to develop them. There is potential for MMF to streamline the MI-BE process for LAIs enabling smaller or shorter BE studies. In small group discussions, members commented that MMFs may need to be thought of as more of a platform, than a specific model for a specific product, so that it can be broadly applied. The MMF framework may also be of use for technology transfers, site changes, and post-approval changes.

## Conclusion

This perspective describes certain opportunities of using MMFs for LAI products, including case studies and potential situations in which the MMF concept can support drug development and regulatory submissions. The implementation of MMFs for regulatory use is at a preliminary stage. Further discussion and collaboration among stakeholders are needed to develop and advance the best practices for implementing MMFs.
