# Correction: Leveraging Model Master Files for Long-Acting Injectables

**DOI:** 10.1007/s11095-025-03832-4

**Published:** 2025-02-12

**Authors:** Yuqing Gong, Robert Hopefl, Tonglei Li, Andrew C. Hooker, Daniela Amaral Silva, Khondoker Alam, Murray Ducharme, Rebecca Moody, Pratik Saha, Andrew Babiskin

**Affiliations:** 1 Division of Quantitative Methods and Modeling, Office of Research and Standards, Office of Generic Drugs, Center for Drug Evaluation and Research, U.S. Food and Drug Administration, 10903 New Hampshire Avenue, Silver Spring, MD 20993 USA; 2 Department of Industrial and Physical Pharmacy, Purdue University, West Lafayette, IN USA; 3 Department of Pharmacy, Uppsala University, Uppsala, Sweden; 4 Simulations Plus, Inc, Lancaster, CA USA; 5 Learn and Confirm Inc, St. Laurent, QC Canada; 6 Office of Pharmaceutical Quality, Center for Drug Evaluation and Research, U.S. Food and Drug Administration, Silver Spring, MD USA; 7 GlaxoSmithKline, Collegeville, PA USA

**Correction: Pharmaceutical Research**

10.1007/s11095-025-03824-4

The original article has been updated to remove the incorrect reference citation "(Lachaine et al Can J Psychiatry. 60:S40-S47, 2015)” from the first sentence in the abstract. This error was introduced in production and was not the fault of the authors.

